# Progress of Research on Exosomes in the Protection Against Ischemic Brain Injury

**DOI:** 10.3389/fnins.2019.01149

**Published:** 2019-10-29

**Authors:** Xianhui Kang, Ziyi Zuo, Wandong Hong, Hongli Tang, Wujun Geng

**Affiliations:** ^1^Department of Anesthesiology, The First Affiliated Hospital of Wenzhou Medical University, Wenzhou, China; ^2^Department of Anesthesiology, The First Affiliated Hospital, College of Medicine, Zhejiang University, Hangzhou, China; ^3^The First Clinical College, Wenzhou Medical University, Wenzhou, China; ^4^Department of Gastroenterology and Hepatology, The First Affiliated Hospital of Wenzhou Medical University, Wenzhou, China

**Keywords:** exosomes, brain protection, ischemic brain injury, stroke, drug delivery

## Abstract

Exosomes, as a type of extracellular vesicle (EV), are lipid bilayer vesicles 20–100 nm in diameter that can cross the blood-brain barrier. Exosomes are important transport vesicles in the human body that participate in many conduction pathways and play an important physiological role. Because of their high biocompatibility and low immunogenicity and toxicity, exosomes have attracted increasing attention as an attractive drug delivery system. This article reviews the relevant studies that have shown that exosomes play an important role in protective mechanisms against ischemic brain injury.

## Introduction

For ischemic brain injury, pharmacological and non-pharmacological brain protection methods are commonly used in the clinic. Pharmacological methods include ion channel blockers, lipid peroxidation inhibitors, excitatory amino acid (EAA) antagonists, blood sugar reduction, barbiturates, and traditional Chinese medicine, whereas non-pharmacological methods include mild hypothermia treatment and acupuncture. Research has examined both ischemic postconditioning and ischemic preconditioning.

In recent years, an increasing number of studies have shown that exosomes can act on the central nervous system through crossing the blood-brain barrier due to their own properties and contents and protect brain tissues through various mechanisms; these findings suggest that exosomes from various sources can protect the brain through cerebral ischemic preconditioning and ameliorate nervous system diseases in the clinic. Exosomes are derived from the intracellular lysosome pathway. Intracellular lysosome particles invade and form multivesicular bodies (MVBs). Then, the extracellular membrane of these vesicles fuses with the cell membrane and secretes them to the extracellular matrix (Colombo et al., 2014). Exosomes, which are between 20 and 100 nm in diameter, are important transport vesicles that can cross the blood-brain barrier and participate in multiple signaling pathways. Exosomes play an important role in the normal physiological function of cells and the occurrence and development of diseases, but research on exosomes is relatively new. Exosomes have been found to mediate the occurrence and development of related diseases such as Alzheimer’s disease and Parkinson’s disease by participating in the production, secretion, aggregation and uptake of related “toxic” proteins, suggesting that exosomes may be an important marker for the early diagnosis of related diseases. This article reviews the latest progress of research on exosomes in the field of ischemic brain injury protection.

## Overview of Exosomes

### Discovery of Exosomes

Pan and Johnstone (1983) studied the transformation of sheep reticulocytes to mature erythrocytes *in vitro*. Through ultracentrifugation, a small vesicle was isolated from the supernatant of sheep erythrocytes. Under electron microscopy, the vesicle was found to be composed of a lipid bilayer with a round or concave cup-like structure and was later named an exosome. For some time afterward, exosomes were considered carriers of waste transported by cells to the outside world. In Raposo et al. (1996) discovered that B lymphocyte-derived exosomes have multiple functions, including antigen presentation, T lymphocyte activation, and immune cell function regulation. Related functions of exosomes began to be discovered gradually. After further study, exosomes were found to be widely present in human blood, cerebrospinal fluid, saliva, urine and so on. In Valadi et al. (2007) discovered for the first time that exosomes contained both RNA and microRNA and confirmed that the RNA carried by exosomes had certain biological activities. With the gradual discovery of substances carried by exosomes, the important roles of proteins, lipids and RNA carried by exosomes in intercellular information exchange and genetic material transfer have increasingly become hot research subjects in the fields of disease occurrence, disease treatment and disease prevention.

### Biogenesis and Composition of Exosomes

Extracellular vesicles (EVs) include exosomes with a diameter of 20–100 nm, microvesicles with a diameter of 20–1000 nm and apoptotic bodies with a diameter of 500–2000 nm. Exosomes originate from the endolysosome pathway, whereas microvesicles originate from the direct germination of cells, making the composition of microvesicles much simpler than that of exosomes. The exosome formation process mainly includes early endosomal formation by invagination of the cytoplasmic membrane and early endosomal formation by regulation of the endosomal sorting complex (ESCRT) to form multiple intraluminal vesicles (ILVs), which then constitute MVBs. MVBs mature and fuse with lysosomes for lysosome degradation or fuse with plasmalemma, releasing ILVs to the cell surface to form exosomes (Samanta et al., 2018).

**FIGURE 1:**
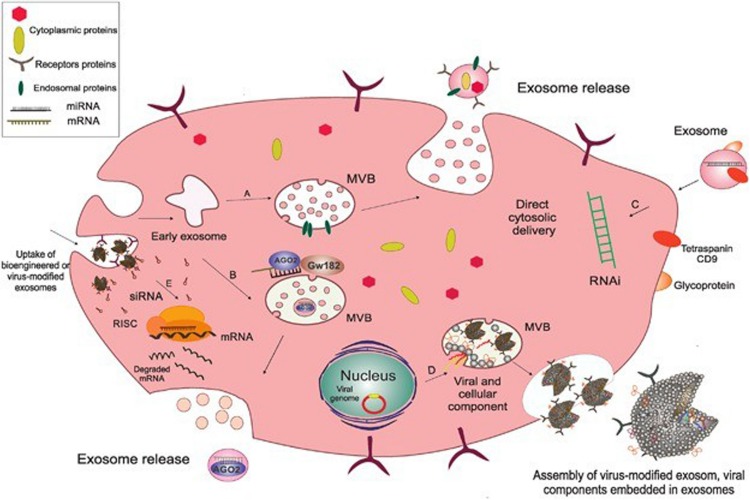
The biogenesis and composition of exosomes. Exosomes originate as endocytic vesicles through invagination of the cell membrane, which results in the formation of early exosomes and, subsequently, late exosome called MVBs. MVBs contain ILV which are formed from budding of the endosomal membrane and are called exosomes. When MVBs fuse with the plasma membrane, they give rise to the release of exosomes into the extracellular space. (A) Endosomal proteins such as CD9, Alix, and TSG101 may be incorporated into exosomes during their assembly to facilitate this process. (B) AGO2 and GW182 are two important components of the RNA-induced silencing complex (RISC), which associate with MVBs so as to mediate miRNA sorting into exosomes. (C) Subsequent fusion of cell-derived exosomes with the plasma membrane through their CD9 (tetraspanin) interaction with surface glycoproteins on target cells gives rise to cytosolic delivery of the siRNA directly. This process is involved in the creation of temporary RNAi. (D) Exosomes originate from inward budding in the lumen of the MVB through which the cytoplasmic content from the cell of origin and also viral components, including mRNA and small non-coding RNA and viral glycoproteins are incorporated into the exosome, and then released as a selective cargo in viral-infected cells. These virus-modified exosomes display the original surface markers and cell membrane as the parent cells. (E) Bioengineered or virus-modified exosomes are designed to express a selective set of proteins and small, non-coding RNAs to target specific receptors. Exosomes then fuse with the endosomal membrane to release their non-coding RNAs into the cytoplasm so as to load siRNA into the RNAi (RISC) complex of the target cells in order to prevent mRNA translation into protein. Acronyms: RISC, RNA-induced silencing complex; RNAi, RNA interference; siRNA, small interference RNA; MVBs, multivesicular bodies.

The composition of exosomes has been examined by trypsin digestion, mass spectrometry, Western blot and fluorescence-activated cell sorting (FACS). Exosomes are lipid bilayer vesicles rich in cholesterol, ceramide, sphingomyelin and phospholipids with long saturated ester chains. Exosomes contain a variety of proteins: protein membrane transport fusion proteins (GTPases, annexins, flotillin), transmembrane proteins (CD9, CD63, CD81 and CD82), heat shock proteins (Hsp70, Hsp60, Hsp20, Hsp90) (Gupta and Knowlton, 2007; Zhang et al., 2012) and other proteins (Alix, TSG101), lipoproteins and phospholipases (Roucourt et al., 2015) involved in the formation of vesicles. In addition, exosomes contain many microRNAs, RNAs and other non-coding RNAs, which can be transferred between cells and then regulate the expression of related genes (Pegtel et al., 2010). Many scholars are now focusing on the RNA contained in exosomes and its corresponding regulatory role. An increasing number of scholars are examining the mechanisms of exosomes in mediating disease and tissue protection. The biogenesis and composition of exosomes as shown in Figure 1 (Shahabipour et al., 2017).

### Regulation of Exosome Secretion

Precise regulation of exosome secretion is important for various cell functions. The molecular mechanisms that directly regulate exosome secretion have been studied in recent year. Increasing numbers of studies have shown that some essential regulators of exosome biogenesis and secretion in diverse cell types (Hessvik et al., 2016; Wei et al., 2017). Endosomal sorting complexes required for transport proteins (e.g., HRS and Tsg101), tetraspanins (e.g., CD81 and CD9), lipids (e.g., ceramide) and Rab GTPases (e.g., Rab11, Rab27, and Rab35) have been identified to regulate exosome secretion and release (Hsu et al., 2010; Ostrowski et al., 2010; Colombo et al., 2013; Sims et al., 2018). However, the upstream platform for exosome regulators is not well understood. Song L. et al. (2019) revealed that KIBRA controls exosome secretion via inhibiting the proteasomal degradation of Rab27a. Given that Exosomes play a vital role in intercellular communication and numerous biological processes, the exact molecular mechanisms implicated in Exosomes secretion warrant further exploration.

### Purification of Exosomes

Separation of exosomes is the first step in functioning as a carrier, and thus, appropriate separation is the key to maintenance of their physical, chemical, and biological functions. Given the substantial differences in exosome size and surface markers, the methods for separation of exosomes must have high specificity and high efficiency. The commonly used methods of separation include ultracentrifugation, ultrafiltration, precipitation, immunoaffinity procedures and microfluidics (Ayala-Mar et al., 2019).

#### Ultracentrifugation

An effective method for separation of exosomes is the key to the value of exosomes, and therefore, it is essential to reserve the physical, chemical, and biological functions, structure and content of the exosomes to the greatest extent. At present, the golden standard for separation of exosomes is ultracentrifugation (Li P. et al., 2017). By utilizing the differences in the sedimentation rates of components of different molecular weights in a homogeneous suspension and by increasing the centrifugal force gradually, this technique separates cells, cell debris, vesicles, and proteins of different molecular weights and thus purifies exosomes. Distinguishing exosomes, small vesicles and some proteins following ultracentriguation is difficult. Purification is usually achieved by sucrose density gradient centrifugation combined with ultracentrifugation (Gupta et al., 2018).

#### Ultrafiltration

Ultrafiltration allows EVs to pass or remain on a selective membrane based on their different sizes through application of different forces, thereby achieving the purpose of isolating the exosomes of a specific size.

#### Precipitation

Exosomes are precipitated by mixing the sample with a highly hydrophilic polymer to change the solubility or dispersibility of the exosomes. Polyethylene glycol (PEG) is commonly used for this process and has extensive applications, including the extraction of exosomes from serum, plasma, ascites, and urine.

#### Immunoaffinity Procedures

Immunoblotting based on immunoaffinity is an effective means for identifying the separated exosomes. In addition, immunoaffinity techniques can be used to selectively separate exosomes in complex liquid environments. Exosomes separated using this method have high quality and purity. At present, the magnetic beads are coated with monoclonal antibody microparticles and then specifically bound to the exosome surface proteins to achieve the separation.

#### Microfluidics

Microfluidic techniques, an emerging separation method, include immunoaffinity, screening, and porous structure capture (Liga et al., 2015). Because this method requires a much smaller volume of samples and reagents than other methods, these techniques can complete the processing of small samples in a short period of time and thus have been widely applied in biomedicine, analytical chemistry and other fields.

### Relevance of Exosomes for Occurrence and Development of Diseases

Related studies have discovered that exosomes contain many miRNAs, mRNAs, and other non-coding RNAs, and therefore, as a new form of intercellular communication, these molecules play a very important role in the information transfer between cells (Salem and Fan, 2017). In recent years, the role of exosomes in the development of various diseases has been discovered gradually. For their biological effects, exosomes transfer information from the original cells to the recipient cells mainly through the information transfer between cells and simultaneously release the encoded information into the intercellular fluid or blood circulation, thereby inducing corresponding changes in the recipient cells. Therefore, the occurrence of many diseases is closely related to exosomes. In the course of diabetes development, a variety of miRNAs carried by exosomes, including miR-155 and miR-204, can facilitate the occurrence of diabetes by causing insulin resistance, reducing the sensitivity of the body to insulin, and activating mitochondrial apoptosis in β cells.

Among the exosome-mediated diseases related to the central nervous system, Alzheimer’s disease has been extensively studied. Dinkins et al. found that astrocyte-derived exosomes can aggravate cognitive dysfunction by enriching and blocking the degradation of Aβ42 as a component of the senile plaques of Alzheimer’s disease (Dinkins et al., 2016). Moreover, microglia can internalize and release Tau protein through exosomes. Exosomes can carry overphosphorylated Tau protein into peripheral cells, causing damage to the functions of cells when intracellular regulatory functions are dysfunctional.

Exosomes also play a vital role in the development of various blood-related diseases. In recent years, many studies have found that exosomes are closely linked to hypertension (Pironti et al., 2015), atherosclerosis (Moreno et al., 2013), cardiac hypertrophy and other diseases and can carry and transfer miR-21-3p, miR-133b and other miRNAs, playing an important role in the occurrence and development of the above cardiovascular diseases.

### Advantages of Exosomes as a Natural Carrier System

As an important barrier to isolate plasma and cerebrospinal fluid, the blood-brain barrier plays a crucial role in preventing harmful substances from entering the brain and maintaining the basic stability of the brain environment. However, the restriction of the transport of macromolecule proteins by the blood-brain barrier makes entering the brain through the blood-brain barrier difficult for some macromolecule drugs that would be otherwise effective for the treatment of nervous system diseases, thereby limiting their clinical application. To enable these drugs to be used effectively in the clinic, an effective carrier system is needed to participate in the delivery of drugs.

**FIGURE 2:**
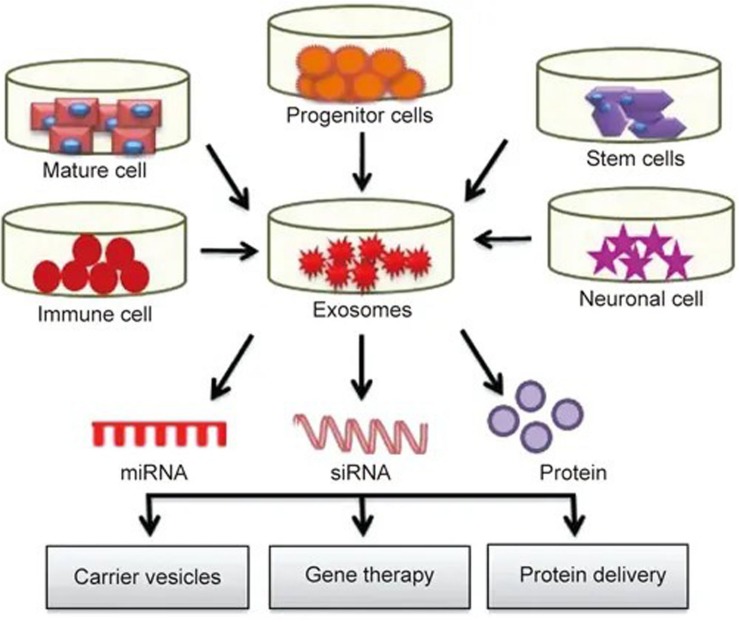
Exosomes play roles in drug delivery: exosomes isolated from different cell types are rich in miRNA, RNA and protein. These molecules can further modified and reinserted into the exosomes for different therapeutic applications.

Currently, carrier systems including liposomes and nanoparticles are widely used, but their high immunogenicity, low biocompatibility, short half-life and lack of specificity are limiting. As a natural carrier system, exosomes have a low immunogenicity, high biocompatibility, long half-life (Ha et al., 2016), and strong targeting ability (Lakhal and Wood, 2011). Exosomes can freely cross the blood-brain barrier (Zhuang et al., 2011) and maintain high activity during long-term storage, giving them major advantages as an ideal drug delivery system. A large number of studies have shown that exosomes can deliver different pharmacological molecules to target cells or tissues. These molecules can be further modified and reinserted into exosomes for different therapeutic applications (see Figure 2), opening up a new method for clinical drug delivery for central nervous system diseases (Samanta et al., 2018).

### Brain Tissue Protection and Treatment in Central Nervous System Diseases

A series of studies have shown that exosomes play a therapeutic role in ischemic diseases of the central nervous system, creating treatment options. Exosomes have a variety of sources (endothelial cells, adipose tissue-derived mesenchymal stem cells, astrocytes, etc.) and can protect against and repair ischemic central nervous system injury. A key characteristic of exosomes is their ability to penetrate the blood-brain barrier and release the associated RNA, protein, etc. into the central nervous system (Valadi et al., 2007) and then pass through it. There are many pathways that promote the growth and repair of blood vessels, inhibit the apoptosis of nerve cells, and promote the repair and regeneration of nerve cells. Long et al. (2017) found that the adult exudate of mesenchymal stem cells administered through the nasal spray administration route can be absorbed by neurons and microglia in the motor cortex, thereby alleviating neuronal inflammation, indicating that exosomes can penetrate the blood-brain barrier and play a therapeutic role in relevant regions of the brain. Among all the sources of exosomes, mesenchymal stem cell-derived exosomes have been extensively studied for their ability to promote the protection and repair of the central nervous system. The neuroprotective effect of mesenchymal stem cell-derived exosomes was found to be related to their dose and number of generations. The smaller the generation, the stronger neuroprotective effect of the exosomes is. Low-dose exosomes could inhibit neuronal injury through antiapoptotic effects and oxidation, while high-dose exosomes had the opposite effect on neurons (Venugopal et al., 2017).

### Protective and Reparative Effects of Exosomes on Brain Tissue Injury

Relevant studies on the protection and repair of brain tissue mediated by exosomes have shown that exosomes can protect and repair neurons by (1) improving the microenvironment and regulating the corresponding immune function; (2) inhibiting neuronal apoptosis and mediating axon reconstruction and neurogenesis; (3) promoting vascular regeneration and remodeling; and (4) alleviating inflammation. Exosomes also play a role in the sexual response and immunosuppression. Dosage and route of administration of exosomes in animal experiments (see Table 1).

**TABLE 1:** Dosages and routes of administration of exosomes in animal experiments.

**Administration route**	**Protein dosage**	**Quantity**	**References**
Intranasal (IN)	15 μg	7.5 × 10^9^ exosomes	Long et al., 2017
Intranasal (IN)	10 μg	–	Kalani et al., 2016
Tail vein injection (TV)	100 μg	3 × 10^9^ exosomes	Zhang et al., 2017c
Intravenous injection (IV)	250 μL	EVs released by 2 × 10^6^ MSCs	Doeppner et al., 2015
Tail vein injection (TV)	100 μg	–	Xin et al., 2013
Tail vein injection (TV)	100 μg/day for 3 days	–	Song Y. et al., 2019
Tail vein injection (TV)	100 μg	–	Shen et al., 2018

#### Improving the Microenvironment and Regulating Immune-Mediated Tissue Protection and Repair

In acute brain injury, insufficient cerebral perfusion can lead to ischemic stroke. White et al. (2000) showed that during stroke, high levels of glutamate accumulate in cells, which opens voltage-dependent and glutamate-regulated calcium channels, resulting in a substantial calcium influx and activation and production of large quantities of nitric oxide synthase. Excessive consumption of superoxide dismutase (SOD) leads to an accumulation of free radicals in cells, causing cell apoptosis, DNA damage, and brain tissue damage. Wei et al. (2016) showed that in a glutamate-induced neuronal injury model, adipose-derived mesenchymal stem cell-derived exosomes can protect brain tissue from glutamate-induced neuronal injury by transporting and releasing cytokines such as insulin-like growth factor (IGF) and hepatocyte growth factor (HGF), which are potentially associated with activation of the phosphatidylinositol 3-kinase (PI3K)/Akt pathway. In addition, Kalani et al. (2016) found that exosomes could reduce the infarct volume and degree of edema in the brain of ischemia-reperfusion mice by reducing the expression of the glutamate receptor *N*-methyl-D-aspartate receptor (NMDAR) in the central nervous system.

Neurons, as permanent cells, are difficult to repair after damage. Although neural stem cells provide hope for regeneration of neurons through self-differentiation, this process is very difficult due to the poor peripheral microenvironment of damaged tissues. Han et al. (2019) showed that intravenous injection of mesenchymal stem cell-derived exosomes into a intracerebral hemorrhage rat model improved the brain microenvironment through crossing the blood-brain barrier and promoting vascular remodeling and neurological function by regulating angiogenesis and neuron regeneration. These results indicated that mesenchymal stem cell-derived exosomes could enter the brain microenvironment and improve it to promote the repair of damaged neurons.

#### Promoting Vascular Regeneration and Reconstruction

The protective effect of exosomes is also reflected in their ability to promote the regeneration and reconstruction of blood vessels. Du et al. (2018) co-cultured mesenchymal stem cell-derived exosomes with high expression of microRNA-132-3p and bEnd.3 with mouse brain microvascular endothelial cells damaged by glucose and oxygen deprivation/reoxygenation (H/R). The mesenchymal stem cell-derived exosomes expressing high levels of microRNA-132-3p effectively improved the proliferation and migration function of H/R-induced damaged cerebrovascular endothelial cells and reduced Akt phosphorylation levels, thereby promoting blood vessel regeneration through the PI3K/Akt pathway. These observations indicated that exosomes can promote the regeneration of cerebrovascular endothelial cells by promoting the proliferation of cerebrovascular endothelial cells, providing a new method for stem cell therapy for the treatment of cerebrovascular injury.

#### Inhibiting Neuronal Apoptosis and Mediating Axon Remodeling and Neurogenesis

Shen et al. (2018) showed that the number of apoptotic and degenerative neurons in the rat brain was significantly reduced by transfecting exosomes with microRNA-133b and transfusing them back into the rat tail vein after intracerebral hemorrhage, indicating that microRNA-133b-containing exosomes had a protective effect on the brain tissue after intracerebral hemorrhage. Similarly, Li et al. (2018) found that neural stem cell (NSC)-derived exosomes could inhibit neuronal apoptosis and promote neuronal survival by studying the role of NSC-derived exosomes in a cobalt chloride (CoCl_2_)-induced hypoxia model. Furthermore, Xin et al. (2013) showed that exosomes can promote neuronal axon remodeling, neurogenesis and angiogenesis in stroke models. In addition, culturing neurons and glial cells in the presence of mesenchymal stem cell-derived exosomes expressing abundant microRNA-133b promoted neuronal cell growth, indicated by an increase in neuron neurites (Xin et al., 2012). Similarly, Zhang et al. (2017b) found that exosomes derived from mesenchymal stem cells promoted axon formation in nerve cells, which may be related to microRNA-17-92. Song Y. et al. (2019) observed mouse infarction and neuronal apoptosis 3 days after an ischemic attack induced by blocking the middle cerebral artery of the mouse, followed by immediate intravenous injection of M2 microglia-derived exosomes; M2 BV2-derived exosomes were shown to promote neuronal-mediated neuroprotection via miR-124 and through downregulation of miR-124 target proteins, which inhibited neurological deficits and neuronal apoptosis in the mouse model of stroke, thereby increasing the survival rate of neuronal cells.

#### Antagonizing Immunosuppression and the Inflammatory Response

Li Y. et al. (2017) showed that exosomes of dental pulp mesenchymal stem cells could inhibit the neuroinflammatory response induced by traumatic brain injury. Huang et al. (2018) also showed that under traumatic brain injury, an increase in microglial exosome miRNA-124-3p not only reduced the occurrence of the inflammatory response but also promoted the growth of axons. Similarly, Zhang et al. (2017c) found that neurological function was improved by extracting mesenchymal stem cell-derived exosomes and transfusing them back into a traumatic brain injury (TBI) animal model. The exosomes may act by promoting vascular remodeling and nerve regeneration and alleviating inflammatory reactions. In addition, Doeppner et al. (2015) found that mesenchymal stem cell-derived exosomes not only promoted cerebral vascular regeneration in mice with focal cerebral ischemia but also provided an appropriate external environment for brain remodeling by antagonizing the immunosuppressive response. This study indicated that exosomes from various sources have various mechanisms by which they provide protection of brain tissues after a trauma. Exosomes are able to protect and repair damaged tissues by antagonizing immune and inflammatory responses, promoting neuron regeneration and providing a suitable reconstructed external environment.

### Protection of Brain Tissue Mediated by Secretion of Exosome in Ischemic Preconditioning

As research on the function of exosomes has been performed and our knowledge of their role has deepened, researchers’ views on exosomes have changed. At an early stage, exosomes were considered a medium for cells to discharge waste to the outside world. In recent years, the above studies showed that exosomes play an important role in the protection and repair of brain tissue after brain injury. The research not only suggests that exosomes can be used as a new therapeutic approach with great potential but also that exosomes play an important role in alleviating or even preventing brain tissue damage caused by ischemia and hypoxia. At present, non-pharmacological approaches such as mild hypothermia and ion channel blockers are mostly used to prevent ischemic brain damage in the clinic, as well as other pharmacological approaches such as ion channel blockers. In recent years, an increasing number of researchers have found that exosomes may play an important role in brain protection mediated by ischemic preconditioning.

Ischemic conditioning refers to a process involving short-term blockade and reperfusion of blood flow to activate various endogenous protective mechanisms and alleviate tissue damage. This method is widely used in various cardiovascular operations. However, because the protection of brain tissue using this process requires the separation of the brain tissue for blood flow blockage and reflux, the process has many advantages. In recent years, the concept of ischemic conditioning has been extended to remote ischemic conditioning (RIC), i.e., a short series of blood flow blockade and reperfusion in the distal limbs through cuff suppression, which has also been found to have protective effects on brain tissue after multiple cycles. Although ischemic preconditioning and RIC have great potential for development as effective, low-cost and simple methods, few researchers have studied the mechanism of ischemic preconditioning.

Xiao et al. (2017) studied the protective effect of RIC in acute cerebral ischemia. RIC on the limbs of experimental animals increased the content of exosomes in the blood, the morphology of which was similar to that of endothelial cell-derived exosomes. Further experiments showed that endothelial cell-derived exosomes could be induced by upregulation of transcription and translation through sugar deprivation/reoxygenation in the SH-SY5Y nerve cell line. In this process, the expression of Bcl-2 inhibits the expression of Bax, thus alleviating nerve cell apoptosis and achieving a protective effect. The mechanism of this process may be related to the Janus kinase 2 (JAK2)/signal transducer and activator of transcription-3 (STAT3) pathway (Cheng et al., 2014) and the PI3K/Akt pathway (Zhang et al., 2017a); the latter pathway has been extensively studied, and the former requires further testing.

In Xiao et al. (2017), the CD63, HSP70 and TSG101 expression levels in exosomes in the hippocampus of the RIP group did not increase, but the expression in plasma increased, indicating that RIP can promote the release of exosomes. This finding indicates that in light of the spatial distribution of exosomes in a model of acute cerebral ischemia, exosomes are extensively distributed in the blood circulation. Moreover, the brain protection mediated by remote ischemic preconditioning also indicates that exosomes are extensively distributed in the whole body through the blood circulation, and due to the size of the exosomes themselves and the specificity of their physical and chemical properties, these molecules can further mediate brain protection by passing through the BBB and releasing miRNAs and other substances. Furthermore, based on the elevated blood exosomes induced by remote ischemic preconditioning, these molecules are likely associated with the protection of other organs. The dependent interaction of exosomes in the damage of organism has been outlined above. In recent year, increasing numbers of studies have shown that in addition to the brain protection mediated by exosomes, exosomes can act on tissues such as the myocardium in a similar manner and mediate the corresponding tissue protective functions, indicating that the effect of exosomes is not specific to brain tissue. Given the diversity of these mechanisms, multiple organ protective mechanisms may be present simultaneously, and further exploration of these mechanisms is needed.

## Outlook

As a new therapeutic carrier, exosomes have attracted increasing attention because of their unique biological characteristics. Our understanding of exosomes has also changed from an excreta carrier in earlier years to a new therapeutic carrier with tremendous research potential in recent years, with an ability to mediate the repair process of multiple brain tissue injuries. Many studies have shown that exosomes can ameliorate ischemic and hypoxic brain tissue by improving the microenvironment, regulating the corresponding immune effects, inhibiting neuronal apoptosis, mediating axon reconstruction and neurogenesis, promoting vascular regeneration and remodeling, alleviating the inflammatory response and immune suppression, etc. Moreover, these repairing effects of exosomes also suggest that they can play a protective role in preventing ischemic brain necrosis by improving the resistance of brain tissue to acute ischemic injury. Through the study of animal models of acute ischemia, we see that RIC can produce exosomes and transfer them to brain tissue to play a protective role, not only indicating that exosomes can play a protective role in preventing ischemic brain necrosis but also showing tremendous research value in the study of their involvement in mediating brain protection. Research has also shown that exosomes, with their high biocompatibility, low immunogenicity and toxicity, can effectively participate in brain protection. In addition to traditional non-pharmacological approaches such as mild hypothermia and pharmacological approaches, exosomes can protect against cerebral ischemia injury. At the same time, the protective effect mediated by exosomes found in remote ischemic preconditioning experiments indicates that exosomes are related to the traditional ischemic preconditioning mechanism.

However, our knowledge of the protective effect of exosomes on brain tissue is limited; most experiments are limited to the protective effect of exosomes on the tissue itself, and the interaction between exosomes and signaling pathways is not discussed in detail. In addition to studies on the use of exosomes as an effective therapeutic approach, more research examining the specific mechanism of exosome-mediated brain tissue protection is needed.

## Author Contributions

WG and HT were the guarantor of integrity of the entire study. WG and WH contributed to study concepts. XK and WH contributed to manuscript preparation. XK and ZZ contributed to manuscript editing. HT helped to manuscript review.

## Conflict of Interest

The authors declare that the research was conducted in the absence of any commercial or financial relationships that could be construed as a potential conflict of interest.

## References

[B1] Ayala-Mar S. Donoso-Quezada J. Gallo-Villanueva R. C. Perez-Gonzalez V. H. Gonzalez-Valdez J. (2019). Recent advances and challenges in the recovery and purification of cellular exosomes. *Electrophoresis* 10.1002/elps.201800526 [Epub ahead of print]. 31373715 PMC6972601

[B2] Cheng Z. Li L. Mo X. Zhang L. Xie Y. Guo Q. (2014). Non-invasive remote limb ischemic postconditioning protects rats against focal cerebral ischemia by upregulating STAT3 and reducing apoptosis. *Int. J. Mol. Med.* 34 957–966. 10.3892/ijmm.2014.1873 25092271 PMC4152138

[B3] Colombo M. Moita C. van Niel G. Kowal J. Vigneron J. Benaroch P. (2013). Analysis of ESCRT functions in exosome biogenesis, composition and secretion highlights the heterogeneity of extracellular vesicles. *J. Cell Sci.* 126 5553–5565. 10.1242/jcs.128868 24105262

[B4] Colombo M. Raposo G. Thery C. (2014). “Biogenesis, secretion, and intercellular interactions of exosomes and other extracellular vesicles,” in *Annual Review of Cell and Developmental Biology*, Vol. 30 eds Schekman R. Lehmann R. (California, CA: Annual Reviews), 255–289. 10.1146/annurev-cellbio-101512-122326 25288114

[B5] Dinkins M. B. Enasko J. Hernandez C. Wang G. Kong J. Helwa I. (2016). Neutral sphingomyelinase-2 deficiency ameliorates Alzheimer’s Disease pathology and improves cognition in the 5XFAD Mouse. *J. Neurosci.* 36 8653–8667. 10.1523/jneurosci.1429-16.2016 27535912 PMC4987436

[B6] Doeppner T. R. Herz J. Goergens A. Schlechter J. Ludwig A.-K. Radtke S. (2015). Extracellular vesicles improve post-stroke neuroregeneration and prevent postischemic immunosuppression. *Stem Cells Transl. Med.* 4 1131–1143. 10.5966/sctm.2015-2078 26339036 PMC4572905

[B7] Du D. Wang Y. Xu X. Zheng J. Zhang H. Kuang X. (2018). Improving effect of exosomes of mesenchymal stem cells with high expression of miR-132-3p on hypoxia/reoxygenation impaired brain microvascular endothelial cell function. *Chin. J. Cerebrovasc. Dis.* 15 584–591.

[B8] Gupta S. Knowlton A. A. (2007). HSP60 trafficking in adult cardiac myocytes: role of the exosomal pathway. *Am. J. Physiol. Heart Circ. Physiol.* 292 H3052–H3056. 10.1152/ajpheart.01355.2006 17307989

[B9] Gupta S. Rawat S. Arora V. Kottarath S. K. Dinda A. K. Vaishnav P. K. (2018). An improvised one-step sucrose cushion ultracentrifugation method for exosome isolation from culture supernatants of mesenchymal stem cells. *Stem Cell Res. Ther.* 9:180. 10.1186/s13287-018-0923-920 29973270 PMC6033286

[B10] Ha D. Yang N. Nadithe V. (2016). Exosomes as therapeutic drug carriers and delivery vehicles across biological membranes: current perspectives and future challenges. *Acta Pharm. Sin. B* 6 287–296. 10.1016/j.apsb.2016.02.001 27471669 PMC4951582

[B11] Han Y. Seyfried D. Meng Y. Yang D. Schultz L. Chopp M. (2019). Multipotent mesenchymal stromal cell-derived exosomes improve functional recovery after experimental intracerebral hemorrhage in the rat. *J. Neurosurg.* 131 290–300. 10.3171/2018.2.jns171475 30028267

[B12] Hessvik N. P. verbye A. Brech A. Torgersen M. L. Jakobsen I. S. Sandvig K. (2016). PIKfyve inhibition increases exosome release and induces secretory autophagy. *Cell. Mol. Life Sci.* 73 4717–4737. 10.1007/s00018-016-2309-2308 27438886 PMC11108566

[B13] Hsu C. Morohashi Y. Yoshimura S. I Manrique-Hoyos N. Jung S. Lauterbach M. A. (2010). Regulation of exosome secretion by Rab35 and its GTPase-activating proteins TBC1D10A-C. *J. Cell Biol.* 189 223–232. 10.1083/jcb.200911018 20404108 PMC2856897

[B14] Huang S. Ge X. Yu J. Han Z. Yin Z. Li Y. (2018). Increased miR-124-3p in microglial exosomes following traumatic brain injury inhibits neuronal inflammation and contributes to neurite outgrowth via their transfer into neurons. *FASEB J.* 32 512–528. 10.1096/fj.201700673R 28935818

[B15] Kalani A. Chaturvedi P. Kamat P. K. Maldonado C. Bauer P. Joshuaa I. G. (2016). Curcumin-loaded embryonic stem cell exosomes restored neurovascular unit following ischemia-reperfusion injury. *Int. J. Biochem. Cell Biol.* 79 360–369. 10.1016/j.biocel.2016.09.002 27594413 PMC5067233

[B16] Lakhal S. Wood M. J. A. (2011). Exosome nanotechnology: an emerging paradigm shift in drug delivery exploitation of exosome nanovesicles for systemic in vivo delivery of RNAi heralds new horizons for drug delivery across biological barriers. *Bioessays* 33 737–741. 10.1002/bies.201100076 21932222

[B17] Li B. Wei H. Yang Y. Ying M. Hu C. Lu Y. (2018). Neural stem cell-derived exosomes inhibit apoptosis of neurons induced by hypoxia neural cells. *Chinese J. Pathophysiol.* 34 717–722, 728.

[B18] Li P. Kaslan M. Lee S. H. Yao J. Gao Z. (2017). Progress in exosome isolation techniques. *Theranostics* 7 789–804. 10.7150/thno.18133 28255367 PMC5327650

[B19] Li Y. Yang Y.-Y. Ren J.-L. Xu F. Chen F.-M. Li A. (2017). Exosomes secreted by stem cells from human exfoliated deciduous teeth contribute to functional recovery after traumatic brain injury by shifting microglia M1/M2 polarization in rats. *Stem Cell Res. Ther.* 8:198. 10.1186/s13287-017-0648-645 28962585 PMC5622448

[B20] Liga A. Vliegenthart A. D. B. Oosthuyzen W. Dear J. W. Kersaudy-Kerhoas M. (2015). Exosome isolation: a microfluidic road-map. *Lab Chip* 15 2388–2394. 10.1039/c5lc00240k 25940789

[B21] Long Q. Upadhya D. Hattiangady B. Kim D.-K. An S. Y. Shuai B. (2017). Intranasal MSC-derived A1-exosomes ease inflammation, and prevent abnormal neurogenesis and memory dysfunction after status epilepticus. *Proc. Natl. Acad. Sci. U.S.A.* 114 E3536–E3545. 10.1073/pnas.1703920114 28396435 PMC5410779

[B22] Moreno J. A. Sastre C. Madrigal-Matute J. Munoz-Garcia B. Ortega L. Burkly L. C. (2013). HMGB1 expression and secretion are increased Via TWEAK-Fn14 interaction in atherosclerotic plaques and cultured monocytes. *Arterioscler. Thromb. Vasc. Biol.* 33 612–620. 10.1161/atvbaha.112.300874 23288170

[B23] Ostrowski M. Carmo N. B. Krumeich S. Fanget I. Raposo G. Savina A. (2010). Rab27a and Rab27b control different steps of the exosome secretion pathway. *Nat. Cell Biol.* 12 19–30. 10.1038/ncb2000 19966785

[B24] Pan B. T. Johnstone R. M. (1983). Fate of the transferrin receptor during maturation of sheep reticulocytes invitro - selective externalization of the receptor. *Cell* 33 967–977. 10.1016/0092-8674(83)90040-90045 6307529

[B25] Pegtel D. M. Cosmopoulos K. Thorley-Lawson D. A. van Eijndhoven M. A. J. Hopmans E. S. Lindenberg J. L. (2010). Functional delivery of viral miRNAs via exosomes. *Proc. Natl. Acad. Sci. U.S.A.* 107 6328–6333. 10.1073/pnas.0914843107 20304794 PMC2851954

[B26] Pironti G. Strachan R. T. Abraham D. Yu S. M.-W. Chen M. Chen W. (2015). Circulating exosomes induced by cardiac pressure overload contain functional Angiotensin II Type 1 receptors. *Circulation* 131 2120–2130. 10.1161/circulationaha.115.015687 25995315 PMC4470842

[B27] Raposo G. Nijman H. W. Stoorvogel W. Leijendekker R. Harding C. V. Melief C. J. M. (1996). B lymphocytes secrete antigen-presenting vesicles. *J. Exp. Med.* 183 1161–1172. 10.1084/jem.183.3.1161 8642258 PMC2192324

[B28] Roucourt B. Meeussen S. Bao J. Zimmermann P. David G. (2015). Heparanase activates the syndecan-syntenin-ALIX exosome pathway. *Cell Res.* 25 412–428. 10.1038/cr.2015.29 25732677 PMC4387558

[B29] Salem E. S. B. Fan G.-C. (2017). “Pathological effects of exosomes in mediating diabetic cardiomyopathy,” in *Exosomes in Cardiovascular Diseases: Biomarkers, Pathological and Therapeutic Effects*, eds Xiao J. Cretoiu S. (Berlin: Springer), 113–138. 10.1007/978-981-10-4397-0_8 PMC6205207 28936736

[B30] Samanta S. Rajasingh S. Drosos N. Zhou Z. Dawn B. Rajasingh J. (2018). Exosomes: new molecular targets of diseases. *Acta Pharmacol. Sin.* 39 501–513. 10.1038/aps.2017.162 29219950 PMC5888687

[B31] Shahabipour F. Barati N. Johnston T. P. Derosa G. Maffioli P. Sahebkar A. (2017). Exosomes: nanoparticulate tools for RNA interference and drug delivery. *J. Cell. Physiol.* 232 1660–1668. 10.1002/jcp.25766 28063231 PMC7166392

[B32] Shen H. Yao X. Li H. Li X. Zhang T. Sun Q. (2018). Role of exosomes derived from miR-133b modified mscs in an experimental rat model of intracerebral hemorrhage. *J. Mol. Neurosci.* 64 421–430. 10.1007/s12031-018-1041-1042 29455449

[B33] Sims B. Farrow A. L. Williams S. D. Bansal A. Krendelchtchikov A. Matthews Q. L. (2018). Tetraspanin blockage reduces exosome-mediated HIV-1 entry. *Arch. Virol.* 163 1683–1689. 10.1007/s00705-018-3737-3736 29429034 PMC5958159

[B34] Song L. Tang S. Han X. L. Jiang Z. Y. Dong L. L. Liu C. C. (2019). KIBRA controls exosome secretion via inhibiting the proteasomal degradation of Rab27a. *Nat. Commun.* 10:1639. 10.1038/s41467-019-09720-x 30967557 PMC6456494

[B35] Song Y. Li Z. He T. Qu M. Jiang L. Li W. (2019). M2 microglia-derived exosomes protect the mouse brain from ischemia-reperfusion injury via exosomal miR-124. *Theranostics* 9 2910–2923. 10.7150/thno.30879 31244932 PMC6568171

[B36] Valadi H. Ekstrom K. Bossios A. Sjostrand M. Lee J. J. Lotvall J. O. (2007). Exosome-mediated transfer of mRNAs and microRNAs is a novel mechanism of genetic exchange between cells. *Nat. Cell Biol.* 9 654–659. 10.1038/ncb1596 17486113

[B37] Venugopal C. Shamir C. Senthilkumar S. Babu J. V. Sonu P. K. Nishtha K. J. (2017). Dosage and passage dependent neuroprotective effects of exosomes derived from rat bone marrow mesenchymal stem cells: an in vitro analysis. *Curr. Gene Ther.* 17 379–390. 10.2174/1566523218666180125091952 29366415

[B38] Wei J. J. Chen Y. F. Xue C. L. Ma B. T. Shen Y. M. Guan J. (2016). Protection of nerve injury with Exosome extracted from mesenchymal stem cell. *Zhongguo Yi Xue Ke Xue Yuan Xue Bao* 38 33–36. 10.3881/j.issn.1000-503X.2016.01.006 26956853

[B39] Wei Y. Wang D. Jin F. Bian Z. Li L. Liang H. (2017). Pyruvate kinase type M2 promotes tumour cell exosome release via phosphorylating synaptosome-associated protein 23. *Nat. Commun.* 8:14041. 10.1038/ncomms14041 28067230 PMC5228053

[B40] White B. C. Sullivan J. M. DeGracia D. J. O’Neil B. J. Neumar R. W. Grossman L. I. (2000). Brain ischemia and reperfusion: molecular mechanisms of neuronal injury. *J. Neurol. Sci.* 179 1–33. 10.1016/s0022-510x(00)00386-385 11054482

[B41] Xiao B. Chai Y. Lv S. Ye M. Wu M. Xie L. (2017). Endothelial cell-derived exosomes protect SH-SY5Y nerve cells against ischemia/reperfusion injury. *Int. J. Mol. Med.* 40 1201–1209. 10.3892/ijmm.2017.3106 28849073 PMC5593464

[B42] Xin H. Li Y. Buller B. Katakowski M. Zhang Y. Wang X. (2012). Exosome-mediated transfer of mir-133b from multipotent mesenchymal stromal cells to neural cells contributes to neurite outgrowth. *Stem Cells* 30 1556–1564. 10.1002/stem.1129 22605481 PMC3495063

[B43] Xin H. Li Y. Cui Y. Yang J. J. Zhang Z. G. Chopp M. (2013). Systemic administration of exosomes released from mesenchymal stromal cells promote functional recovery and neurovascular plasticity after stroke in rats. *J. Cereb. Blood Flow Metab.* 33 1711–1715. 10.1038/jcbfm.2013.152 23963371 PMC3824189

[B44] Zhang W. Wang Y. Bi G. (2017a). Limb remote ischaemic postconditioning-induced elevation of fibulin-5 confers neuroprotection to rats with cerebral ischaemia/reperfusion injury: activation of the Akt pathway. *Clin. Exp. Pharmacol. Physiol.* 44 656–663. 10.1111/1440-1681.12742 28251683

[B45] Zhang Y. Chopp M. Liu X. S. Katakowski M. Wang X. Tian X. (2017b). Exosomes derived from mesenchymal stromal cells promote axonal growth of cortical neurons. *Mol. Neurobiol.* 54 2659–2673. 10.1007/s12035-016-9851-9850 26993303 PMC5028236

[B46] Zhang Y. Chopp M. Zhang Z. G. Katakowski M. Xin H. Qu C. (2017c). Systemic administration of cell-free exosomes generated by human bone marrow derived mesenchymal stem cells cultured under 2D and 3D conditions improves functional recovery in rats after traumatic brain injury. *Neurochem. Int.* 111 69–81. 10.1016/j.neuint.2016.08.003 27539657 PMC5311054

[B47] Zhang X. Wang X. Zhu H. Kranias E. G. Tang Y. Peng T. (2012). Hsp20 functions as a novel Cardiokine in promoting angiogenesis via activation of VEGFR2. *PLoS One* 7:e32765. 10.1371/journal.pone.0032765 22427880 PMC3299679

[B48] Zhuang X. Xiang X. Grizzle W. Sun D. Zhang S. Axtell R. C. (2011). Treatment of brain inflammatory diseases by delivering exosome encapsulated anti-inflammatory drugs from the nasal region to the brain. *Mol. Ther.* 19 1769–1779. 10.1038/mt.2011.164 21915101 PMC3188748

